# Integrated transcriptomic and functional characterization of Claudin-1 reveals its oncogenic and immunomodulatory roles in pancreatic ductal adenocarcinoma

**DOI:** 10.1080/21688370.2026.2648150

**Published:** 2026-04-14

**Authors:** Anju Surendranath, Yogain Taank, Saira Hamid, Tharini Karthikeyan, Ikhlak Ahmed, Imadeldin Elfaki, Rashid Mir, Rakesh Kumar, Sameer Mirza, Mayank Singh, Shahab Uddin, Ammira S. Al-Shabeeb Akil, Muzafar A. Macha, Punita Dhawan, Ajaz A. Bhat

**Affiliations:** a Metabolic and Mendelian Disorders Clinical Research Program, Precision OMICs Research & Translational Science, Sidra Medicine, Doha, Qatar; b Department of Surgery and Cancer Biology, University of Kansas Medical Center, Kansas City, KS, USA; c Watson-Crick Center for Molecular Medicine, Islamic University of Science and Technology, Kashmir, India; d Department of Biochemistry, Faculty of Science, University of Tabuk, Tabuk, Saudi Arabia; e Department of Medical Laboratory Technology, Prince Fahad Bin Sultan Chair for Biomedical Research, Faculty of Applied Medical Sciences, University of Tabuk, Tabuk, Saudi Arabia; f School of Biotechnology, Shri Mata Vaishno Devi University, Katra, India; g Department of Chemistry, College of Science (COS), United Arab Emirates University (UAEU), Al Ain, United Arab Emirates; h Department of Medical Oncology, Dr. B.R. Ambedkar Institute Rotary Cancer Hospital, All India Institute of Medical Sciences, New Delhi, India; i Translational Research Institute, Academic Health System, Hamad Medical Corporation, Doha, Qatar; j Dermatology Institute, Academic Health System, Hamad Medical Corporation, Doha, Qatar

**Keywords:** Claudins, differentially expressed genes, epithelial-mesenchymal transition, genomic aberrations, immune infiltration, Kaplan-Meier survival plots, methylation, pancreatic ductal adenocarcinoma

## Abstract

Pancreatic ductal adenocarcinoma (PDAC) remains among the deadliest malignancies, driven by its invasive nature and lack of effective biomarkers. Disruption of the epithelial barrier, mediated by tight junction components, is a critical yet underexplored contributor to PDAC progression. Claudins, integral regulators of tight junction integrity, display altered expression across cancers, but their prognostic and immunomodulatory roles in PDAC remain unclear. We performed an integrative analysis of 177 RNA-Seq datasets from TCGA and GTEx to characterize Claudin family alterations in PDAC. Differential expression, copy number variation, methylation, and co-expression networks were analyzed alongside clinical and survival data. Prognostic significance was assessed using Kaplan – Meier and Cox regression analyses, while immune cell infiltration was examined using deconvolution algorithms. Functional validation of Claudin-1 was conducted in Capan-1 cells using CRISPR/Cas9 knockout, followed by proliferation, wound-healing, and Western blot assays. Ten Claudin genes were significantly dysregulated, with Claudin-1 and Claudin-4 frequently amplified and associated with advanced stage and poor survival. High Claudin-1 expression correlated with reduced immune infiltration, indicating an immune-excluded phenotype characterized by immune cells retained in the tumor stroma but largely absent from the tumor parenchyma. Claudin-1 knockout markedly inhibited proliferation, migration, and EMT, evidenced by downregulation of Snail and Slug and restoration of E-cadherin expression. This integrative transcriptomic and functional study identifies Claudin-1 as a key driver of PDAC aggressiveness and immune modulation. These findings establish Claudin-1 as a promising prognostic biomarker and therapeutic target for restoring epithelial integrity and counteracting immune evasion in pancreatic cancer.

## Introduction

Pancreatic cancer (PC) represents a challenge to global health, standing as one of the leading causes of mortality among various cancers.^1^ According to GLOBOCAN 2022 estimates, pancreatic cancer ranks 12th in global incidence with 510,992 new cases (≈2.6% of all cancers) and 6th in cancer-related mortality with 467,409 deaths (≈4.8% of all cancer deaths) worldwide.^2^ The age-standardized (world) incidence and mortality rates are 4.7 and 4.2 per 100,000, respectively. In the United States, pancreatic cancer continues to carry a very poor prognosis: recent statistics report an age-adjusted incidence rate of 13.8 per 100,000 and a death rate of 11.3 per 100,000, with an overall 5-year relative survival of ~13.3%.^3^ The disease predominantly affects older adults, with a median age at diagnosis of approximately 71 years, and incidence peaking in the 65–79 age range.^3^ In 2025, it is estimated that there will be 67,440 new cases and 51,980 deaths in the United States.^3^

The distinct subtypes of pancreatic adenocarcinoma include pancreatic ductal adenocarcinoma (PDAC) and pancreatic acinar cell carcinoma (PACC), both of which pose challenges due to differences in cellular origin, biological behavior, and therapeutic response.^4,5^ Among pancreatic malignancies, PDAC is responsible for about 90% of cases,^6^ and is characterized by its aggressive nature and resistance to conventional therapies, contributing to its poor prognosis.^7^ The risk factors include a range of influences, such as male sex, dark skin, and advancing age. Additional risk factors include environmental and lifestyle variables (such as obesity and any history of tobacco use) and comorbidities (diabetes mellitus, chronic pancreatitis, and *Helicobacter pylori* infection). Hereditary PC genes and specific genetic cancer syndromes also contribute to the total risk profile, indicating that the genetic landscape also plays a part in the progression of PDAC.^8,9^ The treatment for PDAC typically depends on the cancer stage, the patient’s overall health, and other individual factors.^10^ Surgical treatment, such as the Whipple procedure, distal pancreatectomy, or total pancreatectomy, may be considered for localized tumors.^11^ However, due to the often late-stage diagnosis, surgery is not always feasible. Chemotherapy, either adjuvant (post-surgery) or palliative (for advanced cases), plays a central role in systemic PC control.^12^ Targeted therapies, such as erlotinib or nab-paclitaxel, may be utilized in combination with chemotherapy.^13^ Despite these efforts, the overall prognosis for PDAC remains challenging. The limited success in improving PDAC outcomes is due to its late diagnosis, early metastasis, and resistance to available treatment modalities. Therefore, recognizing these challenges is essential for a deeper understanding of the molecular mechanisms driving PDAC development and progression.

The aggressive behavior and unfavorable prognosis of PDAC pose a significant challenge in cancer research. Beyond epidemiology, PDAC is fundamentally an epithelial disease in which loss of tissue organization and microenvironmental rewiring occur alongside changes in epithelial barrier integrity. Tight junctions are apical intercellular complexes that form a selective paracellular barrier and maintain apico – basal polarity (“fence” function). In addition to regulating permeability, tight junctions organize signaling proteins that influence epithelial proliferation, differentiation, and stress responses – processes that progressively dysregulate during tumor evolution.^14^

We prioritized claudins because they are the primary transmembrane determinants of tight-junction structure and function, and their dysregulation can couple barrier failure to cancer-relevant signaling programs rather than merely reflecting a passive consequence of transformation. In the pancreas, tight junctions of the ductal epithelium are integral to normal physiology, and inflammatory mediators and carcinogens can trigger tight-junction disassembly and pancreatic barrier dysfunction – events discussed as relevant to pancreatic disease progression, including pancreatic cancer.^15^ Disruptions in the epithelial barrier, a critical defense against tumor cell dissemination, have emerged as key drivers of PDAC progression.^16^ Among the regulators of this barrier, the Claudin family has attracted attention, with aberrant expression implicated in various cancers, including PDAC.^17^ The Claudins in humans comprise a set of integral membrane proteins that are crucial for forming tight junctions, maintaining epithelial cell integrity, and regulating paracellular permeability.^18^ Encoded by various genes, including claudin-1 (*CLDN1)*, claudin-2*(CLDN2)*, claudin-3*(CLDN3)*, claudin-4*(CLDN4)*, claudin-5*(CLDN5)*, claudin-6*(CLDN6)*, claudin-7*(CLDN7)*, claudin-8*(CLDN8)*, claudin-9*(CLDN9)*, claudin-10*(CLDN10)*, claudin-11*(CLDN11)*, claudin-12*(CLDN12)*, claudin-13*(CLDN13)*, claudin-14*(CLDN14)*, claudin-15*(CLDN15)*, claudin-16*(CLDN16)*, claudin-17*(CLDN17)*, and claudin-18(*CLDN18)*, these proteins contribute to the network of cell-cell interactions.^19^ Claudins play diverse roles in several physiological processes, including the selective passage of ions and solutes across epithelial barriers.^20^ However, their dysregulation is associated with pathological conditions, including cancer, where alterations in Claudin expression can impact tumor progression and metastasis.^21^ The dynamic interplay of Claudin human genes underscores their significance in maintaining tissue homeostasis and their potential as therapeutic targets for various disorders. Existing studies have revealed the diverse roles of individual Claudins in PDAC.^22^ Claudin-18.2, for instance, exhibits frequent overexpression, portending a grim prognosis and a higher propensity for metastasis.^22^ Moreover, Claudin expression has been linked to an intriguing counterpoint: high immune cell infiltration within the tumor microenvironment, potentially due to increased tumor immunogenicity.^23^ However, this infiltration often coincides with an immunosuppressive microenvironment, hindering effective anti-tumor immune responses. While previous studies have highlighted the roles of individual Claudins in PDAC, an understanding of their collective impact on prognosis and their correlation with the TME remains elusive.

This study retrieved the RNA-seq datasets from the TCGA and GTEx databases.^24,25^ Clinical and survival information was obtained from cBioPortal, and the relevant clinical variables were merged from both TCGA PanCancer and TCGA Firehose Legacy datasets. The focus was explicitly on PDAC, ensuring a targeted exploration of this cancer type. Subsequently, genomic aberrations were extracted to reveal the frequency and distribution of mutations and copy number variations (CNVs) in Claudin genes. The differences in Claudin expression between the tumor and normal groups were also evaluated using log2FC and gene *p*-values. The study also examined genomic and epigenomic associations, investigating correlations between Claudin expression and genetic and epigenetic factors. Co-expression analysis further revealed functional interactions among Claudin genes, while correlations with clinical parameters demonstrated their clinical relevance. Survival analysis, incorporating Cox regression and Kaplan-Meier plots, evaluated the predictive value of Claudin genes. Further, the association between immune cell infiltration and Claudin expression highlighted the potential interactions within the TME. Additionally, *CLDN1* knockdown in the Capan-1 pancreatic cancer cell line inhibited cell proliferation, wound healing, and EMT.

## Methodology

### Data acquisition and preprocessing

Gene expression data was retrieved from the UCSC Xena platform using UCSCXenaTools in R programming software.^26,27^ Specifically, The Cancer Genome Atlas (TCGA) TOIL RNA-seq recomputed dataset was obtained, offering RSEM-quantified TPM values for samples containing TCGA, TARGET, and GTEX cohorts.^24,25^ Clinical and survival information was sourced from cBioPortal, and the relevant clinical variables were merged from both TCGA PanCancer and TCGA Firehose Legacy datasets. To ensure robust data consistency, log-transformed TPM values initially expressed in the unit of log2 (tpm +0.001) were uniformly adjusted to log2 (tpm +1). Each sample was annotated with its clinical indication before merging with the expression dataset. Ultimately, a pancreatic cancer dataset was extracted from the merged data to focus our analysis on this specific cancer type.

## Genomic aberration and expression analysis

The frequency of genomic aberrations associated with claudin genes was investigated using the R package “maftools” on TCGA Pan-Cancer 2018 data.^28^ This package provides a landscape of Claudin mutations and CNV aberrations across all TCGA-PAAD patients by generating an oncoplot. Further, we contrasted the Claudin expression level of Claudins between normal and tumor groups. The tumor group comprised TCGA-PAAD samples, whereas the normal group incorporated matched normal samples from TCGA-PAAD and GTEX pancreatic samples. To identify dysregulated Claudins in pancreatic cancer, we applied stringent criteria: log2FC ≥ 1 and statistically significant *p*-values, with Bonferroni correction for multiple testing.^29^ The log2FC ≥ 1 threshold was selected to capture biologically meaningful changes in gene expression while retaining sufficient sensitivity to detect moderately but consistently dysregulated Claudin genes, which may be functionally relevant in PDAC. More stringent cutoffs (e.g., log2FC ≥ 2) were not used to avoid excluding potentially important genes with moderate expression changes that could still contribute to tumor biology. Moreover, the Wilcoxon rank-sum test served as the statistical workhorse for comparing tumor and normal groups, with a *p*-value threshold of *p* < 0.05 indicating statistical significance. The resulting *p*-values were categorized into distinct significance levels for clarity (ns: *p* ≥ 0.05; *p* < 0.05; *p* < 0.01; *p* < 0.001). This explores Claudin genomic aberrations and their impact on gene expression in pancreatic cancer.

## Identification of genomic and epigenomic associations

The association between differentially expressed Claudins and their corresponding genomic and epigenomic data was determined using Pearson’s correlation coefficient. The interrelation between matched mRNA expressions and log2 copy number values for each significant Claudin was identified. Similarly, we investigated the association between mRNA levels and hm27/hm450 methylation levels for the respective Claudins, exploring potential epigenetic regulation of Claudin expression. The integration of genomic and epigenomic data revealed a correlation between genetic and epigenetic factors in modulating claudin’s expression. This identifies Claudin regulation at the molecular level, providing valuable insights for future research and potential treatments.

## Co-expression analysis of claudin genes

We further conducted an in-depth co-expression analysis to understand the functional interactions among the identified Claudins. The correlation analysis employed Pearson’s correlation coefficient, a robust statistical measure, with thresholds determined by the relationship between Claudin expression patterns. A rho (*p*) ≥0.5 and *p* < 0.05 threshold was utilized for Claudins exhibiting potential co-expression. Moreover, a distinct threshold for Claudins, indicating potential mutual exclusivity (rho (*p*) <−0.5 and *p* < 0.05), was chosen. This stringent criterion identifies situations in which Claudin expression patterns exhibit a strong negative correlation, implying a mutually exclusive relationship in their functional roles. This helps clarify the roles of these fundamental molecular components.

## Correlation of claudin gene expression with clinical parameters

The clinical relevance of the significant Claudins was explored by associating them with various clinical parameters. The expression levels of Claudin genes were plotted against a spectrum of clinical features, including cancer stage, histological grade, and TNM stage. The Kruskal-Wallis test, a statistical test sensitive to differences between groups, was used to compare expression levels across these distinct clinical categories. This allows us to understand the notable variations of Claudin expression patterns in diverse clinical scenarios, offering valuable insights into the potential clinical implications of these molecular markers in cancer research.

## Survival analysis of claudin genes

To assess the predictive value of Claudin genes for patient outcomes in PAAD, we used Cox regression and Kaplan-Meier survival analysis.^30,31^ In the Cox regression method, univariate and multivariate analyses were conducted using the *survival* and *ezcox* packages in R programming software.^32,33^ This evaluates the independent prognostic value of each Claudin while accounting for potential confounding by other clinical factors. Furthermore, we employed Kaplan-Meier plots to depict the relationship between Claudin expression and patient survival. Patients were divided into low and high-expression groups based on the median expression level of each Claudin gene. This aids in understanding the impact of Claudin expression on overall survival probabilities and potential differences in survival outcomes between the two groups. These informative plots were created using the “survminer” package, while additional statistical analyses were performed with the “survival” package.^34^ A log-rank *p*-value of <0.05 was considered statistically significant, indicating a robust relationship between Claudin expression and survival outcomes.

## Correlation of claudin expression with immune cell infiltration in PDAC

The correlation between Claudin gene expression and immune cell infiltration was assessed to understand the potential interaction between Claudins and the tumor immune microenvironment in PDAC. The focus was on Claudin-1 (*CLDN1*) and Claudin-4 (*CLDN4*), with consideration of their potential implications based on prior analysis. To capture the full spectrum of the immune landscape, we first performed Pearson correlation analyses across the entire TCGA-PDAC cohort. Subsequently, to investigate potential context-dependent effects at extreme expression levels, patients were categorized into low and high expression groups utilizing the top and bottom quartiles (Q1 vs. Q4) as cutoffs. This approach creates a balanced distribution of patients across the groups and facilitates robust analysis.

The immune cell infiltration was estimated within each group using two complementary methods. First, we utilized the “immunedeconv” R package, which employs the “EPIC” deconvolution algorithm. This algorithm infers the relative proportions of different immune cell types based on bulk tumor gene expression profiles. Additionally, we utilized the TIMER 2.0 web server, a widely used resource for analyzing tumor immune infiltrates from TCGA data. This resource provides a range of deconvolution algorithms and comprehensive estimates of immune infiltration across different cancer types. By utilizing both “immunedeconv” and TIMER 2.0, we aimed to enhance the reliability and robustness of our findings.

## Claudin isoform expression in PDAC using single cell analysis

To validate our findings, we utilized the *DISCo (Database of Immune Single-Cell Omics)*, which provides single-cell transcriptomic data across various tissues. This allowed us to assess Claudin isoform expression in pancreatic ductal adenocarcinoma (PDAC) at the single-cell level, enabling a more precise correlation of our results.

### Cell culture and transfection

Capan-1 pancreatic cancer cell line was obtained from American Type Culture Collection (ATCC) and was maintained in ATCC-recommended media supplemented with 10% fetal bovine serum (R&D Systems) and 1% Penicillin-Streptomycin antibiotic (Gibco). Cultures were maintained in a humidified incubator at 37°C and 5% CO_2._ CRISPR Cas9 plasmids targeting *CLDN1* were purchased from Genecopoeia, Inc. (sg1: GAGCGAGTCATGGCCAACGC). Briefly, Capan-1 cells were plated on a 6-well plate containing 2 mL medium per well to attain 70% confluence the next day. Cells were transfected with serum-free media containing Turbofect transfection reagent and 2.0 µg of plasmid. Transfected cells were maintained in a medium containing 3 µg/mL puromycin to select for successfully transfected cells.

### Cell viability assays

To assess the effect of *CLDN1* knockout (KO) on cell proliferation, PrestoBlue (Invitrogen)cell viability assay was performed according to the manufacturer’s instructions. Briefly, 5000 cells/well were seeded in a 96-well plate and incubated for 72 hours. Fluorescence measurements were normalized and analyzed using an unpaired t-test using Welch correction in GraphPad Prism 9.

### Wound healing

Cells were seeded into a 6-well plate and grown until a monolayer was formed. The cells were scratched from the center of the well using a 200 µL sterile micropipette tip. The cells were repeatedly washed with PBS to remove detached cells, then replaced with 2% serum-containing medium. The wounded area was recorded using an inverted microscope at 0, 4, 8, and 16 hours. The wound-healing rate was calculated as follows:

$$\%\mathrm{Wound}\;\mathrm{closure}=\left[\left(\mathrm{Initial}\;\mathrm{wound}\;\mathrm{area}-\mathrm{Final}\;\mathrm{wound}\;\mathrm{area}\right)/\mathrm{Initial}\;\mathrm{wound}\;\mathrm{area}\right]x100$$

## Western blotting

Cells were scraped and lysed in RIPA buffer containing 0.1% protease inhibitor cocktail. Total protein was quantified using Bradford reagent (Bio-Rad). A total of 30 µg protein was loaded per sample and separated by SDS-PAGE (10–12% polyacrylamide gel), then blotted onto PVDF membranes using the wet transfer technique. After blocking with 5% BSA, the membranes were incubated with primary antibodies, washed with TBST, and incubated with HRP-conjugated secondary antibodies, then washed again with TBST. The chemiluminescent blots were developed using Clarity Western ECL and visualized in the Gel Doc Imaging System (Bio-Rad). The list of primary antibodies used in the present study is provided in Supplementary Table S1.

### Statistical analysis

The data presented include at least three biological replicates. Results were expressed as mean ± standard error of the mean (SEM). Experimental data were analyzed using GraphPad Prism version 9. Statistical significance was ascertained within each group via an unpaired t-test followed by Welch’s correction. Results with *p*-values < .05 were considered statistically significant. Significance is denoted using asterisks as follows: *p < 0.05; **p < 0.01; ***p < 0.001; ****p < 0.0001.

Comparisons between two groups were performed using unpaired t-tests with Welch’s correction, while comparisons among multiple groups were conducted using the Kruskal – Wallis test. To address multiple comparisons, appropriate correction methods were applied depending on the analysis. Differential expression analyses between tumor and normal samples were corrected using the Bonferroni method. For correlation analyses, co-expression analyses, and immune infiltration analyses involving multiple testing, false discovery rate (FDR) correction was applied using the Benjamini – Hochberg method.

Survival analyses were conducted using Kaplan – Meier curves with log-rank tests, and Cox proportional hazards regression models were used for univariate and multivariate analyses. A corrected *p*-value of less than 0.05 was considered statistically significant.

## Results

### Genomic aberrations of claudins in pancreatic adenocarcinoma

**Figure 1.**
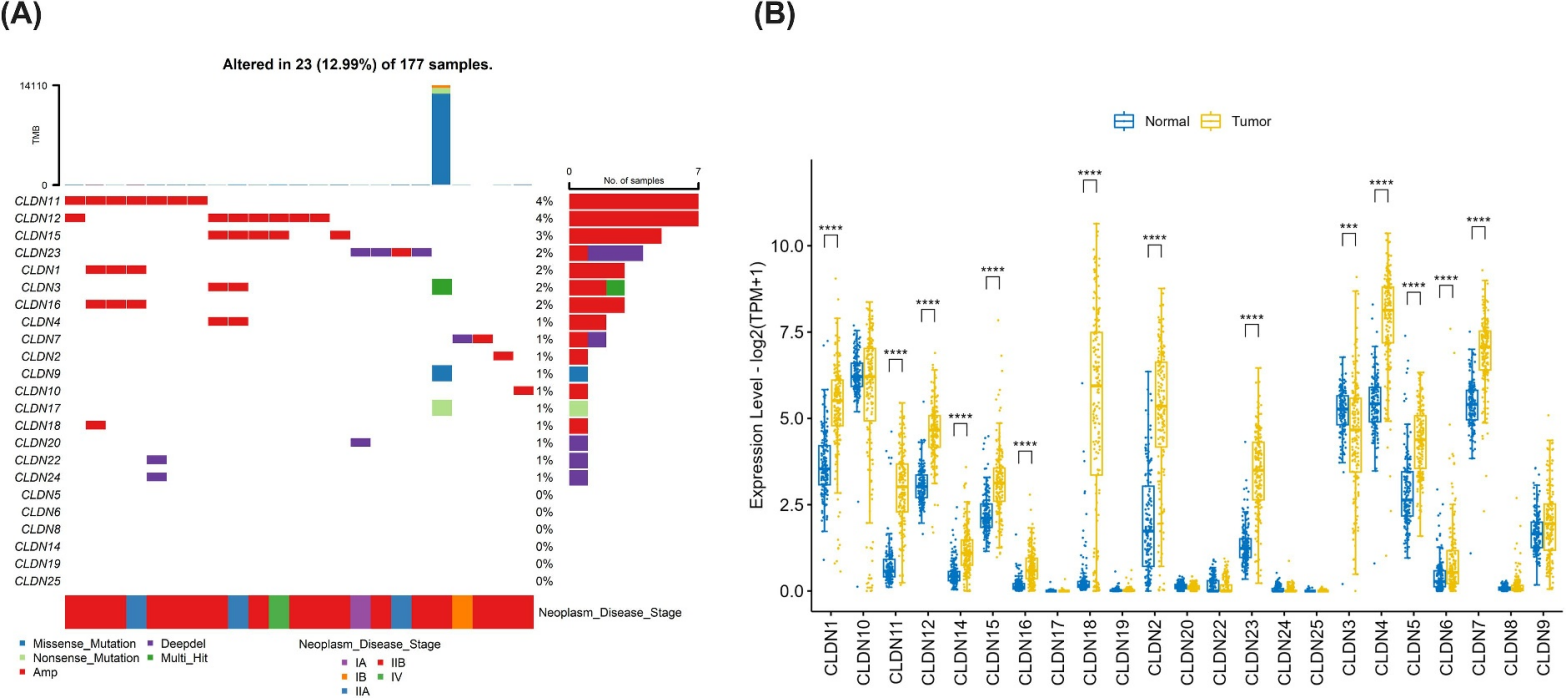
(A) Oncoplot shows the distribution of mutations and copy number variation (CNV) of Claudin genes and the total percentage of patients with these aberrations in the TCGA-PAAD cohort. Across the Claudin family on the Y-axis, 13% of samples exhibit alterations, with rare mutations highlighted in green, observed in only one patient across *CLDN3*, *CLDN9*, and *CLDN17*. Amplifications, the most prevalent genomic aberration, are depicted in red, affecting *CLDN11*, *CLDN12*, *CLDN15*, and *CLDN1* at 4%, 4%, 3%, and 2%, respectively. Additionally, deep deletions highlighted in purple contribute to the genetic diversity of Claudin genes. Overall, this oncoplot provides insight into Claudin gene alterations in pancreatic adenocarcinoma, highlighting the rarity of mutations, the prevalence of amplifications, and the complexity introduced by deep deletions. (B) The set of boxplots presents a detailed comparison of relative Claudin mRNA expression levels in pancreatic adenocarcinoma and normal pancreas tissues, utilizing data from the TCGA tumors with GTEx database controls. The y-axis represents expression levels in log2(TPM +1), while the x-axis shows Claudin family members. Statistical significance, determined by the Wilcoxon rank sum test, is indicated by asterisks (*p < 0.05, **p < 0.01, ***p < 0.001, ****p < 0.0001). Blue boxes represent Claudin mRNA expression in normal samples, while yellow boxes depict expression in pancreatic adenocarcinoma, enabling a visual contrast. The scale facilitates the assessment of relative expression, highlighting differences within the Claudin family. The observed statistical significance shows the clinical relevance of altered Claudin mRNA levels in pancreatic adenocarcinoma.

The analysis investigated the frequency and type of genomic alterations within Claudin genes in a cohort of 177 PDAC samples. Only 13% (23 out of 177) exhibited at least one alteration in a Claudin gene, highlighting the relatively infrequent occurrence of such genomic aberrations in PDAC. Among these alterations, mutations were uncommon, observed in only one patient across *CLDN3*, *CLDN9*, and *CLDN17*. This finding indicates that mutations in Claudin genes play a limited role in PDAC development. Moreover, the amplifications emerged as the most frequent type of genomic aberration, affecting *CLDN11*, *CLDN12*, *CLDN15*, and *CLDN1* at rates of 4%, 4%, 3%, and 2%, respectively (Figure 1(A)). These observations suggest amplification might be a more prevalent mechanism for Claudin dysregulation in PDAC than mutations.

## Differential expression of claudins in pancreatic adenocarcinoma

After retrieving data from The Cancer Genome Atlas (TCGA) and the Genotype-Tissue Expression (GTEx) Project, we analyzed the expression patterns of all Claudin genes in pancreatic adenocarcinoma. This involved a comparative analysis between tumor (*n* = 179) and normal (*n* = 169) groups for each Claudin gene. To ensure robustness, stringent criteria were implemented, requiring a log2 fold change (log2FC) threshold of ≥1 and a statistically significant *p*-value (*p* < 0.05) after Bonferroni correction for multiple testing. The study revealed 10 of the 23 Claudin genes as significantly upregulated in pancreatic adenocarcinoma compared to normal tissues (Figure 1(B)). Detailed information regarding these differentially expressed Claudins, including their log2FC, *p*-values (both unadjusted and adjusted for multiple comparisons), and significance levels, is presented in Table 1.

**Table 1.** Table depicting the detailed information regarding differentially expressed Claudins, including their log2FC, *p*-values (both unadjusted and adjusted for multiple comparisons), and significance levels.

Gene	log2FC	p-value (Wilcoxon rank sum test)	p.adj value (Bonferroni correction)	p.adj.signif
*CLDN1*	2	2.79 × 10^−32^	6.42 × 10^−31^	****
*CLDN11*	2.4	2.50 × 10^−46^	5.75 × 10^−45^	****
*CLDN12*	1.6	6.02 × 10^−47^	1.38 × 10^−45^	****
*CLDN15*	1	5.65 × 10^−24^	1.30 × 10^−22^	****
*CLDN18*	5.8	2.09 × 10^−40^	4.81 × 10^−39^	****
*CLDN2*	3.6	1.93 × 10^−33^	4.44 × 10^−32^	****
*CLDN23*	2.3	9.30 × 10^−44^	2.14 × 10^−42^	****
*CLDN4*	2.7	6.42 × 10^−42^	1.48 × 10^−40^	****
*CLDN5*	1.7	4.48 × 10^−29^	1.03 × 10^−27^	****
*CLDN7*	1.7	4.37 × 10^−38^	1.01 × 10^−36^	****

## Association of claudin expression with copy number and methylation

**Figure 2.**
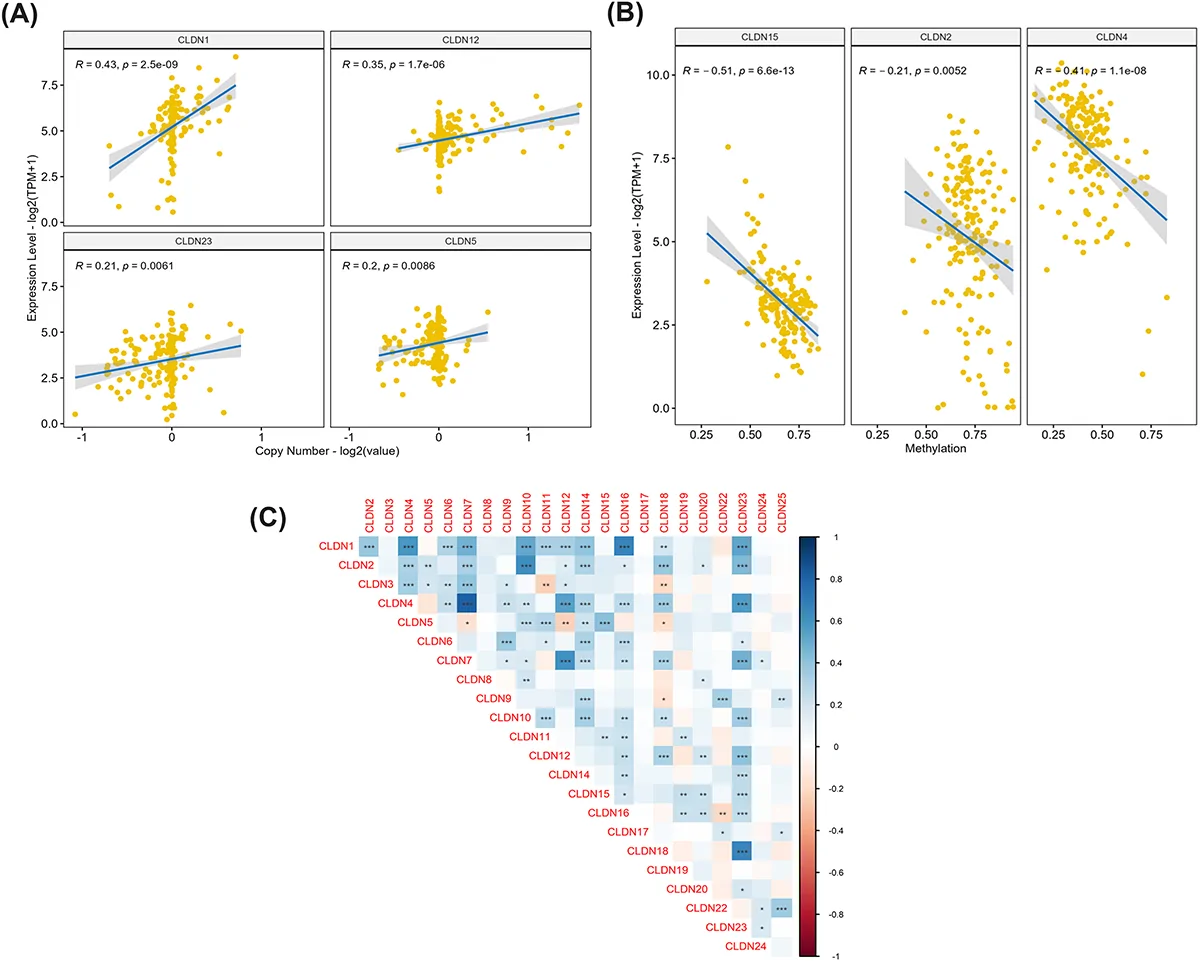
(A) Scatterplots depicting the correlation between Claudin mRNA levels and copy number in pancreatic adenocarcinoma, highlighting Claudins with significant associations. The x-axis represents copy number alterations, while the y-axis showcases each Claudin gene’s corresponding mRNA expression levels. *CLDN1* shows the strongest positive correlation (*p* = 0.43, *p* = 2.48 × 10^−9^), indicating a robust relationship between mRNA expression and copy-number alterations. *CLDN12* (*p* = 0.35, *p* = 1.69 × 10^−6^), *CLDN23* (*p* = 0.21, *p* = 0.006083), and *CLDN5* (*p* = 0.2, *p* = 0.0086) also demonstrate significant positive correlations, underscoring the potential regulatory role of copy number variations in influencing Claudin gene expression. (B) Scatterplots highlighting the correlation between Claudin mRNA levels in pancreatic adenocarcinoma and methylation, signifying the negative associations for *CLDN15* (*p* =  −0.51, *p* = 6.61 × 10^−13^) and *CLDN4* (*p* =  −0.41, *p* = 1.07 × 10^−8^). The x-axis comprehensively represents methylation values, while the y-axis depicts each Claudin gene’s corresponding mRNA expression levels. *CLDN15* and *CLDN4* exhibit negative correlations with methylation levels, suggesting that DNA methylation modulates the expression of these Claudin genes in pancreatic adenocarcinoma. (C) The correlation matrix depicts the interrelationships among Claudins in pancreatic adenocarcinoma by using Pearson’s correlation as the statistical test. The color-coding scheme indicates that dark hues signify high correlation, and lighter shades represent lower correlation (*p < 0.05, **p < 0.01, ***p < 0.001). As depicted in the figure, *CLDN1* demonstrates significant positive co-expression with *CLDN16* (*p* = 0.66, *p* = 2.26 × 10^−24^), *CLDN4* (*p* = 0.59, *p* = 2.05 × 10^−18^), *CLDN23* (*p* = 0.52, *p* = 3.09 × 10^−14^), and *CLDN1*0 (*p* = 0.51, *p* = 1.52 × 10^−13^). *CLDN4* exhibits substantial co-expression with *CLDN7* (*p* = 0.82, *p* = 7.44 × 10^−45^), *CLDN23* (*p* = 0.56, *p* = 1.10 × 10^−16^), and *CLDN12* (*p* = 0.54, *p* = 1.65 × 10^−15^). The significant co-expression is also observed between *CLDN2* and *CLDN10* (*p* = 0.62, *p* = 8.14 × 10^−21^), *CLDN7* and *CLDN12* (*p* = 0.60, *p* = 1.53 × 10^−19^), and *CLDN18* and *CLDN23* (*p* = 0.65, *p* = 1.3 × 10^−23^).

Pearson’s correlation coefficient was used to assess the association between log2-transformed copy number values and mRNA expression levels for each Claudin gene. The results revealed a positive correlation, suggesting that most Claudins showed higher mRNA expression with increasing copy number. These findings align with the observed frequent amplifications of certain Claudins, as amplification events typically lead to increased gene dosage and subsequent higher expression levels. The most significant positive correlations were observed in *CLDN1* (*p* = 0.43, *p* = 2.48 × 10^−9^), *CLDN12* (*p* = 0.35, *p* = 1.69 × 10^−6^), and *CLDN23* (*p* = 0.21, *p* = 0.006083) (Figure 2(A)). Moreover, the analysis investigated the relationship between Claudin expression and methylation levels. Two Claudins, *CLDN15* (*p* = −0.51, *p* = 6.61 × 10^−13^) and *CLDN4* (*p* = −0.41, *p* = 1.07 × 10^−8^) presented a significant negative correlation with methylation levels (Figure 2(B)). The reported *p*-values for methylation – expression correlation analyses were adjusted for multiple comparisons using the false discovery rate (FDR) correction based on the Benjamini – Hochberg method. This suggests that increased methylation might contribute to decreased expression of these specific Claudins and vice versa, potentially through increased or decreased transcriptional silencing.

## Co-expression analysis of claudin genes in PDAC

The functional interactions among differentially expressed Claudins in PDAC were explored using co-expression analysis with Pearson’s correlation coefficient. *CLDN1* exhibited significant positive co-expression with *CLDN16* (*p* = 0.66, *p* = 2.26 × 10^−24^), *CLDN4* (*p* = 0.59, *p* = 2.05 × 10^−18^), *CLDN23* (*p* = 0.52, *p* = 3.09 × 10^−14^), and *CLDN10* (*p* = 0.51, *p* = 1.52 × 10^−13^). Similarly, *CLDN4* was significantly co-expressed with *CLDN7* (*p* = 0.82, *p* = 7.44 × 10^−45^), *CLDN23* (*p* = 0.56, *p* = 1.10 × 10^−16^), and *CLDN12* (*p* = 0.54, *p* = 1.65 × 10^−15^). Additionally, significant co-expression was observed in *CLDN2* with *CLDN10* (*p* = 0.62, *p* = 8.14 × 10^−21^), *CLDN7* with *CLDN12* (*p* = 0.60, *p* = 1.53 × 10^−19^), and *CLDN18* with *CLDN23* (*p* = 0.65, *p* = 1.3 × 10^−23^) (Figure 2(C)).These findings suggest the possible coordinated regulation or shared functional roles among certain Claudin genes in PDAC. For instance, the strong positive co-expression between *CLDN1* and *CLDN4* might indicate their involvement in similar biological processes, particularly tight junction formation and barrier function. Further investigation into the biological implications of these genes’ co-expression patterns could provide valuable insights into their functional roles in PC development and progression.

## Correlation of claudin gene expression with clinical parameters

**Figure 3.**
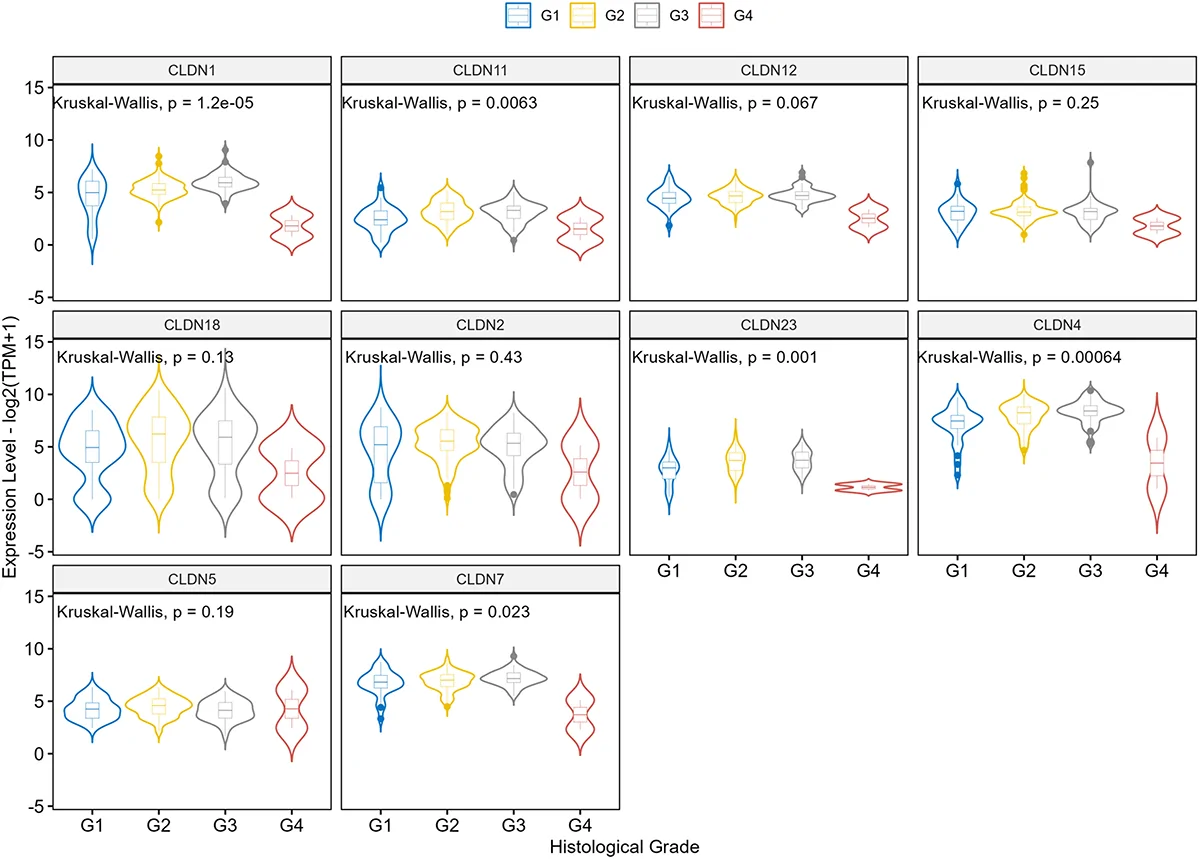
The violin plots show the expression patterns of various Claudin genes across histological grades (G1, G2, G3, and G4) of pancreatic adenocarcinoma, using -log2(TPM +1) as the expression metric. The Kruskal – Wallis test by ranks is employed for statistical analysis. The x-axis represents distinct histological stages, while the y-axis denotes the expression levels of Claudin genes. The figure reveals an inverse relationship between Claudin expression and grade G4 tumors. The expression levels show an upward trend with increasing grade (G2 and G3), particularly for *CLDN1*, *CLDN4*, *CLDN7*, *CLDN11*, *CLDN12*, and *CLDN23*. The pattern suggests a potential role for these Claudins in tumor progression and differentiation processes.

The correlation of Claudin gene expression with various clinical parameters of PDAC patients revealed the clinical relevance of these genes. We analyzed the correlations between Claudin expression levels and cancer stage, histological grade, and TNM stages. The results showed a significant association between Claudin expression and histological grade (Figure 3). The findings demonstrated an inverse relationship between Claudin expression and grade G4 tumors. In contrast, higher grades (G2 and G3) increased expression levels. This pattern was observed across several Claudins, including *CLDN1*, *CLDN4*, *CLDN7*, *CLDN11*, *CLDN12*, and *CLDN23*, suggesting their potential involvement in tumor progression and differentiation.

## Prognostic significance of claudin expression in PDAC

The multivariate and univariate Cox regression analyses revealed the predictive value of Claudin gene expression, along with its relevant clinical characteristics. The univariate analysis highlighted associations between specific Claudins and overall survival. High expression of *CLDN1* and *CLDN4* emerged as significant predictors of poor prognosis, suggesting their potential role in PDAC progression. Conversely, increased expression of *CLDN5* and *CLDN15* was associated with improved prognosis, suggesting a possible tumor-suppressive role. In the multivariate analysis, Claudin expression retained its independent prognostic significance, while other clinical parameters lost their predictive power, highlighting the potential robustness of these associations.

**Figure 4.**
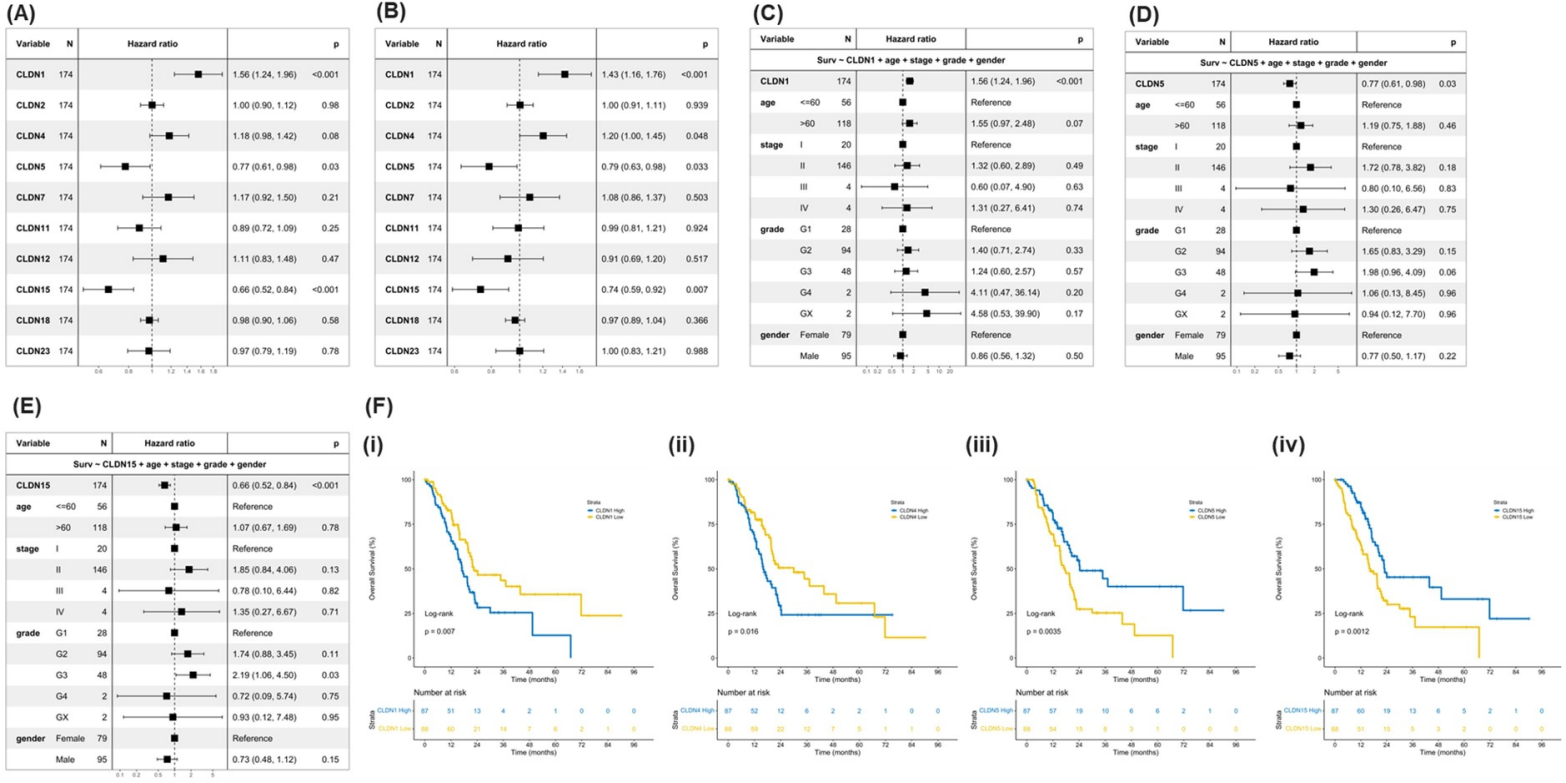
(A) A series of forest plots showcasing the role of Claudin gene expression in influencing pancreatic ductal adenocarcinoma (PDAC) patients’ survival using diverse analytical approaches. Each plot includes individual Claudin genes as variables, presenting the hazard ratio and corresponding *p*-value for each gene. A hazard ratio greater than 1 indicates an increased mortality risk, while a hazard ratio less than 1 suggests a reduced risk. Figure 4A focuses on univariate analysis, highlighting the direct influence of Claudin gene expression on overall survival. (B) Extends this exploration to progression-free survival through univariate analysis, focusing on the disease-free aspect of PDAC progression. (C-E) Delve into multivariate analyses, examining the impact of *CLDN1*, *CLDN5*, and *CLDN15* expressions alongside age, stage, and grade on overall survival. These multivariate analyses offer a more nuanced understanding of the collective influence of Claudin gene expression and clinical parameters on the complex landscape of PDAC patient outcomes. (F) the Kaplan – Meier curves show the overall survival outcomes in distinct high- and low-expression groups for *CLDN1* (i), *CLDN4* (ii), *CLDN5* (iii), and *CLDN15* (iv) in pancreatic adenocarcinoma. The y-axis shows overall survival, and the x-axis shows time in months. The blue line represents the high-expression group, while the yellow line denotes the low-expression group. Statistical significance is determined using *p*-values. In each subfigure, the Kaplan-Meier curves provide the survival divergence between patients with high and low expression of the respective Claudin gene. The time-dependent nature of the analysis allows exploration of variations in Claudin expression and its association with overall survival over months. Hazard ratios (HRs) greater than 1 indicate increased risk of death, whereas HRs less than 1 indicate reduced risk. High- and low-expression groups were defined based on median gene expression levels. Statistical significance was assessed using log-rank tests for Kaplan – Meier analyses and Cox proportional hazards models for regression analyses.

Moreover, the impact of Claudin expression on survival outcomes was elucidated using Kaplan-Meier analysis. The patients were clustered into low and high-expression groups depending on the median expression level of each Claudin. The study confirmed the observed trends, demonstrating that high expression of *CLDN1* and *CLDN4* was associated with significantly lower overall survival. In comparison, high expression of *CLDN5* and *CLDN15* was related to significantly higher survival rates (Figure 4(A–F)). These findings collectively suggest that Claudins can serve as valuable prognostic biomarkers for PDAC patients, potentially aiding risk stratification and guiding individualized patient management strategies.

## Correlation of claudin expression with immune cell infiltration in PDAC

Initial analysis across the complete PDAC cohort suggests a relatively stable, albeit moderate, structural relationship between Claudins and the stroma. Globally, *CLDN1* expression maintains a positive correlation with cancer-associated fibroblast (CAF) infiltration (*p* = 0.23), while *CLDN4* exhibits a modest inverse relationship with endothelial cells (*p* = −0.29) and macrophages (*p* = −0.20). However, these global averages obscure a critical regulatory “switch” that becomes apparent only when examining the extreme expression quartiles (Q1 vs. Q4).

In the low Claudin expression group (Q1), both *CLDN1* and *CLDN4* exhibited positive correlations with specific immune cell populations, suggesting a potential association with enhanced immune infiltration. For *CLDN1*, positive correlations were observed with B cells, cancer-associated fibroblasts (CAF), and CD8+ T cells, suggesting a role in promoting these immune components within the tumor microenvironment. A negative correlation with endothelial cells was also observed for *CLDN1* in this group.

**Figure 5.**
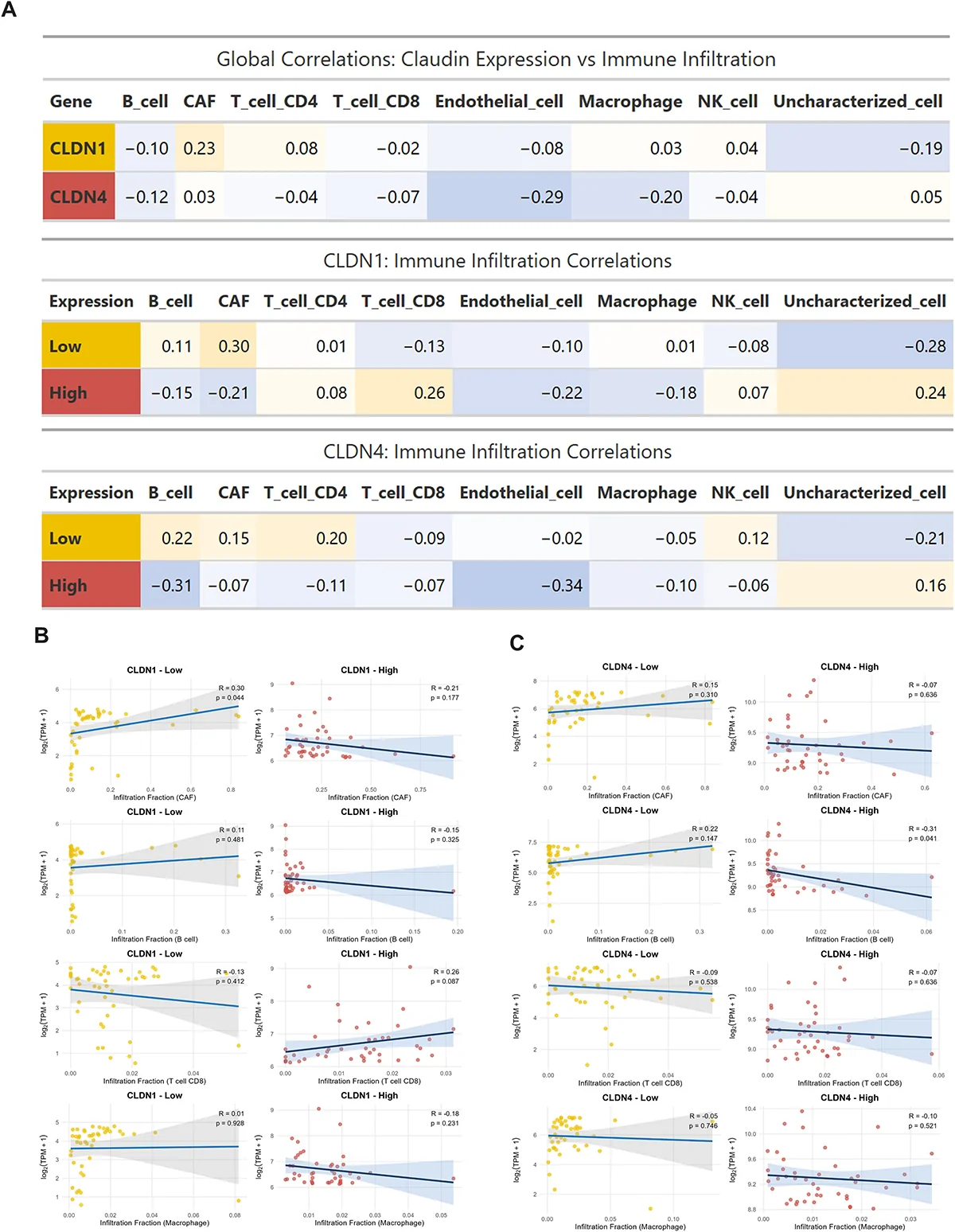
(A) heatmap depicting the correlation coefficients (Pearson’s R) between *CLDN1*/*CLDN4* expression and immune cell proportions. Data are shown for the entire cohort (global) as well as the stratified low (Q1) and high (Q4) expression groups. The immune cell proportions include the B cells, T cells, cancer-associated fibroblasts (CAF), endothelial cells, macrophages, natural killer cells, and other uncharacterized cells. The correlation values are presented separately for the low- and high-CLDN expression groups, providing insight into the relationship between Claudin expression and immune cell infiltration. Positive or negative correlation coefficients signify the strength and direction of the relationship, providing insights into the potential immunomodulatory roles of *CLDN1* and *CLDN4* in the tumor microenvironment. (B)Scatter plots representing the correlation between *CLDN1* expression and various immune cell populations in pancreatic adenocarcinoma between high and low *CLDN1* expression groups. The y-axis represents the expression level of *CLDN1* in terms of -log2(TPM +1), while the x-axis signifies the proportion of cancer-associated fibroblasts (CAF), B cells, CD8 T cells, and macrophages. The figure plots the relationship between *CLDN1* expression and a specific immune cell type, clearly depicting potential associations. The *p*-value for each plot quantifies the statistical significance of the observed correlations. (C) Scatter plots representing the correlation between *CLDN4* expression and various immune cell populations in pancreatic adenocarcinoma between high and low *CLDN4* expression groups. The y-axis represents the expression level of *CLDN4* in terms of -log2(TPM +1), while the x-axis signifies the proportion of cancer-associated fibroblasts (CAF), B cells, CD8 T cells, and macrophages. The figure plots the relationship between *CLDN4* expression and a specific immune cell type, clearly depicting potential associations. The *p*-value for each plot quantifies the statistical significance of the observed correlations.

Conversely, in the high Claudin expression group (Q4), *CLDN1* and *CLDN4* showed negative correlations with most immune cell types, including B cells, CAFs, and endothelial cells, suggesting a potential association with reduced immune infiltration. However, an exception was observed for CD8+ T-cells, where *CLDN1* showed a positive correlation in the high-expression group, hinting at a more complex relationship with this specific immune cell subset (Figure 5(A–C)).These findings suggest the potential context-dependent roles of Claudins in modulating the immune response within the PDAC tumor microenvironment. The observed switch in correlation patterns between low- and high-expression groups suggests that Claudin expression levels maybe a critical determinant of their influence on immune infiltration. Further investigation into the mechanisms underlying these opposing effects could provide insights into the complex relationship between Claudins and the immune system in PDAC, paving the way for new therapeutic strategies that modulate the immune system in this aggressive cancer. An immune-excluded phenotype refers to tumors in which immune cells are present in the surrounding stroma but fail to infiltrate the tumor core, thereby limiting effective tumor – immune interactions.

## Validation with single cell data analysis

**Figure 6.**
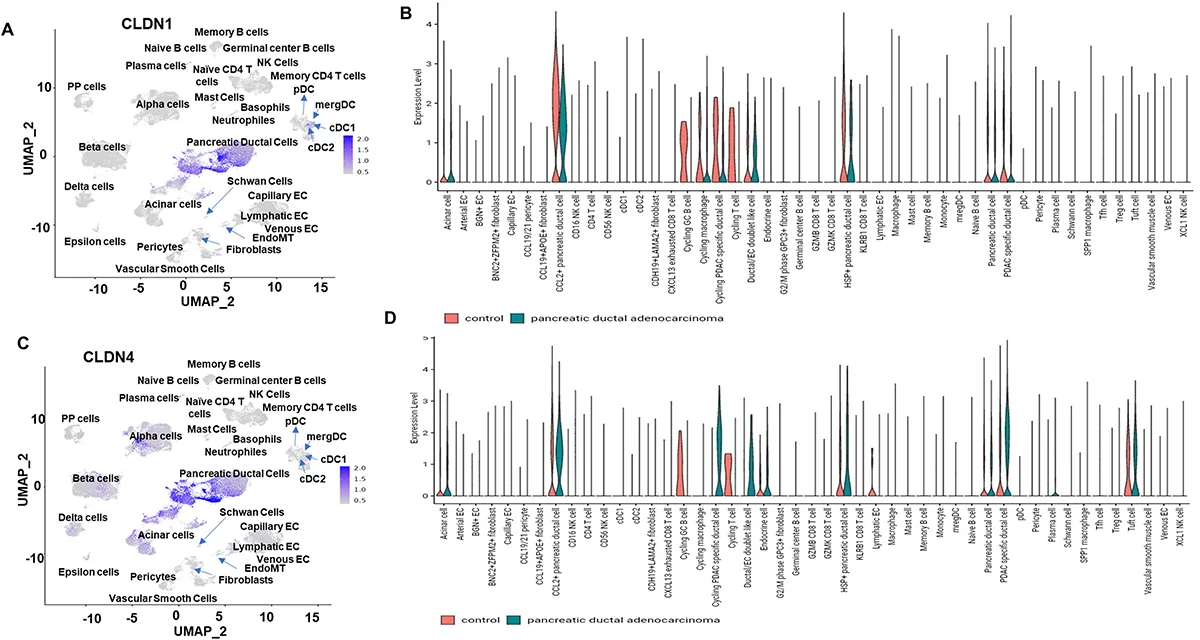
(A)The uniform manifold approximation and projection (UMAP) plot illustrates the expression levels of the *CLDN1* gene across pancreatic cell populations. Cells with higher *CLDN1* expression are represented in blue, while those with lower expression levels are shown in gray. (B) The violin plots illustrate the distribution of *CLDN1* gene expression across various cell populations. The y-axis shows *CLDN1* expression levels, and the x-axis denotes the different cell types analyzed. Red violin plots correspond to control samples, whereas green violin plots represent pancreatic ductal adenocarcinoma (PDAC) samples. (C)The uniform manifold approximation and projection (UMAP) plot illustrates the expression levels of the *CLDN4* gene across pancreatic cell populations. Cells with higher *CLDN4* expression are represented in blue, while those with lower expression levels are shown in gray. (D) The violin plots illustrate the distribution of *CLDN4* gene expression across various cell populations. The y-axis shows *CLDN4* expression levels, and the x-axis denotes the different cell types analyzed. Red violin plots correspond to control samples, whereas green violin plots represent pancreatic ductal adenocarcinoma (PDAC) samples.

To validate our findings, we utilized the DISCO database, which confirmed significant alterations in *CLDN1* expression across multiple pancreatic cell populations. Specifically, differential *CLDN1* expression was observed in acinar cells, cycling macrophages, CCL2+ ductal cells, cycling GC B cells, cycling PDAC-specific ductal cells, cycling T cells, ductal doublet-like cells, pancreatic ductal cells, pancreatic-specific ductal cells, and HSP pancreatic ductal cells (Figure 6(A,B)). This suggests that *CLDN1* expression is dynamically regulated across various cell types, particularly in the tumor microenvironment. The observed changes in cycling immune and ductal cell populations indicate a potential role for *CLDN1* in both tumor progression and immune cell interactions in PDAC. Elevated expression in specific ductal subtypes points toward its involvement in pancreatic epithelial transformation, while its presence in immune cell subsets suggests a role in modulating the immune response. On the other hand, *CLDN4* expression across multiple pancreatic cell populations, including acinar cells, cycling macrophages, CCL2+ ductal cells, cycling GC B cells, cycling PDAC-specific ductal cells, cycling T cells, ductal doublet-like cells, endocrine cells, pancreatic ductal cells, pancreatic-specific ductal cells, lymphatic EC tuft cells, plasma cells, and HSP pancreatic ductal cells (Figure 6(B,C)). The widespread dysregulation of *CLDN4* suggests its potential role in both epithelial transformation and immune modulation within the pancreatic tumor microenvironment. Its altered expression in ductal and acinar cells indicates involvement in PDAC progression. In contrast, changes in immune- and endocrine-related cells suggest broader functional implications, potentially influencing tumor-immune interactions and stromal remodeling. These findings highlight *CLDN4* as a key molecular player in pancreatic cancer and a promising candidate for further investigation as a diagnostic or therapeutic target.

### CLDN1 knockout in pancreatic cancer cell lines reduced cell proliferation and inhibited metastasis

To investigate the role of *CLDN1* in pancreatic cancer aggressiveness, we generated *CLDN1* knockout (KO) Capan-1 cells using a CRISPR/Cas9 shRNA construct, as Capan-1 cells exhibit high endogenous *CLDN1* expression.Capan-1 cells were chosen for functional analysis of *CLDN1* due to their high endogenous *CLDN1* expression, epithelial ductal origin, and molecular alterations commonly observed in pancreatic ductal adenocarcinoma, including activating *KRAS, TP53, CDKN2A, and SMAD4* mutations.^35^ Their stable epithelial phenotype and intact junctional architecture make them a suitable model to investigate the role of in pancreatic cancer – relevant functional outcomes.

**Figure 7.**
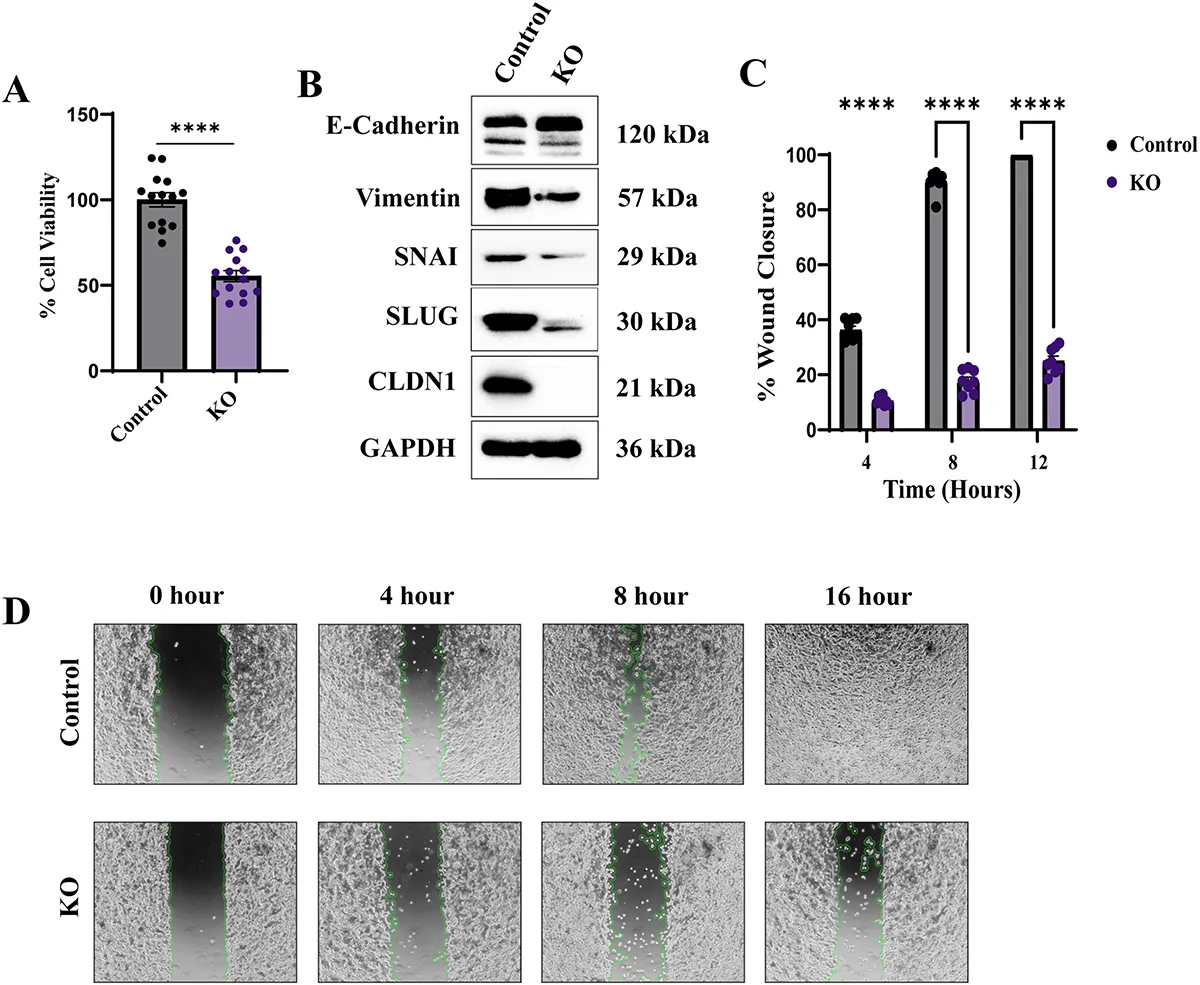
CLDN1 knockout reduces cell proliferation and metastatic potential. A) Loss of *CLDN1* in Capan-1 cells resulted in a significant decrease in cell viability. B) *CLDN1* knockout impaired metastatic potential by suppressing the EMT transcription factors Snail and Slug, leading to downregulation of the epithelial marker E-cadherin and upregulation of the mesenchymal marker vimentin. C) *CLDN1* knockout significantly reduced the migratory capacity of cells. d) Representative photomicrographs of wound closure at different time intervals.

Since *CLDN1* has been implicated in regulating tumor growth and survival, we next sought to evaluate whether its loss impacts the proliferative capacity of pancreatic cancer cells. Inhibition of *CLDN1* expression significantly reduced cell proliferation in KO cells compared with control cells (Figure 7(A)). To further explore the impact of *CLDN1* on metastatic potential, the expression of epithelial – mesenchymal transition (EMT) markers and transcription factors was examined in control and KO cells. Snail (SNAI) and Slug, two key EMT-inducing transcription factors that promote mesenchymal traits by repressing the epithelial marker E-cadherin and inducing mesenchymal markers such as vimentin, were found to be elevated in control cells relative to KO cells. In line with these findings, E-cadherin expression was markedly increased. At the same time, vimentin levels were significantly reduced in KO cells compared with control cells, suggesting that EMT is suppressed upon *CLDN1* loss (Figure 7(B)). To directly assess the impact of *CLDN1* on cell motility, we performed a scratch (wound-healing) assay. Notably, *CLDN1* depletion substantially impaired wound closure in KO cells compared with control cells, indicating a loss of migratory capacity (Figure 7(C,D)). Collectively, these findings demonstrate that *CLDN1* supports proliferative, migratory, and EMT-associated phenotypes, thereby functioning as a key promoter of pancreatic cancer aggressiveness.

## Discussion

PDAC remains one of the most aggressive and fatal malignancies worldwide, ranking among the leading causes of cancer-related mortality.^36^ Despite advances in molecular oncology, the 5-year survival rate for PDAC continues to remain below 10%, primarily due to early metastasis, chemoresistance, and late detection. Among the numerous signaling and structural alterations implicated in PDAC pathogenesis, the disruption of epithelial cell polarity and tight junction integrity has emerged as a key hallmark of tumor progression. The Claudin family of tight junction proteins plays a pivotal role in maintaining epithelial barrier integrity, and their dysregulation has been increasingly recognized in cancer progression, metastasis, and therapeutic resistance.

In this study, we performed an integrative analysis of bulk and single-cell transcriptomic datasets from TCGA and GTEx, coupled with clinical and genomic information from cBioPortal, to investigate the role of Claudin genes in PDAC. Our findings highlight that Claudin gene dysregulation is a frequent event in PDAC, occurring predominantly through copy number amplifications rather than mutations. Although mutations were rare, identified in only 13% of PDAC cases, with a single patient exhibiting alterations in *CLDN3*, *CLDN9*, and *CLDN17*, amplifications in *CLDN1*, *CLDN11*, *CLDN12*, and *CLDN15* were more prevalent (4%, 4%, 3%, and 2%, respectively). These amplifications likely drive aberrant Claudin protein expression, promoting oncogenic signaling, increased proliferation, and metastatic competence.^37–41^ Such amplification-centric regulation aligns with previous evidence that structural components of tight junctions can act as proto-oncogenic molecules when mislocalized or overexpressed in epithelial cancers.^42–44^

Differential expression analysis further identified 10 Claudin genes that were significantly upregulated in PDAC, notably *CLDN1* and *CLDN4*, whose elevated expression correlated with tumor aggressiveness. Prior studies have similarly reported that *CLDN1* and *CLDN4* are overexpressed in pancreatic cancer and contribute to epithelial – mesenchymal transition (EMT), invasiveness, and metastasis.^45,46^ Our findings corroborate these reports, indicating that Claudin overexpression may disrupt junctional homeostasis and enhance signaling cascades that facilitate tumor cell dissemination. Mechanistically, Claudin overexpression may alter paracellular permeability and modulate growth factor receptor clustering, thereby enhancing downstream signaling pathways such as MAPK, AKT, and β-catenin.^42,44,47^ In addition to its role in epithelial – mesenchymal transition (EMT), Claudin-1 overexpression may influence multiple oncogenic signaling cascades that contribute to PDAC progression. Evidence suggests that *CLDN1* can activate the PI3K/AKT pathway, promoting cell survival and resistance to apoptosis, while MAPK/ERK signaling may enhance proliferative and migratory capacities. Furthermore, dysregulated Claudin-1 expression has been associated with Wnt/β-catenin pathway activation, facilitating β-catenin nuclear translocation and transcription of pro-tumorigenic target genes involved in stemness and invasion. Claudin-1 may also alter tight junction integrity in a manner that affects receptor localization and growth factor signaling dynamics, thereby amplifying tumor – stromal crosstalk and contributing to immune exclusion within the tumor microenvironment. Collectively, these pathway-level alterations suggest that Claudin-1 functions not only as a structural tight junction component but also as an active regulator of oncogenic signaling networks in PDAC.

To understand the regulatory mechanisms driving Claudin dysregulation, we examined the associations among copy number variation (CNV), DNA methylation, and mRNA expression. Positive correlations between mRNA expression and CNV for *CLDN1*, *CLDN12*, and *CLDN23* suggest a gene dosage effect contributing to their overexpression. Conversely, the strong negative correlations between promoter methylation and expression for *CLDN15* and *CLDN4* (*p* = −0.51 and −0.41, respectively) indicate that epigenetic silencing may suppress certain Claudins.^48–50^ Such dual genomic and epigenomic regulation underscores the complex transcriptional control of Claudins and may contribute to the heterogeneity observed among PDAC tumors.

The co-expression network revealed strong positive correlations among several Claudin family members, including *CLDN1* with *CLDN16* (*p* = 0.66) and *CLDN4* (*p* = 0.59), and *CLDN4* with *CLDN7* (*p* = 0.82) and *CLDN12* (*p* = 0.54). These findings indicate coordinated regulation and functional interdependence, suggesting that subsets of Claudins may operate within specific signaling modules to modulate barrier function and intercellular communication.^51,52^ Functional redundancy or compensatory mechanisms among Claudins could also explain why individual gene silencing does not always result in complete loss of junctional integrity.

Clinical correlation analysis revealed that high expression of *CLDN1*, *CLDN4*, *CLDN7*, *CLDN11*, *CLDN12*, and *CLDN23* was associated with higher histological grades and advanced TNM stages, reflecting their potential role in tumor progression and dedifferentiation.^53–55^ This association highlights that Claudin expression not only marks the transition from well-differentiated to poorly differentiated tumors but may also directly contribute to invasion and metastasis by weakening cell adhesion. In addition to tumor grade, *CLDN1* expression was also significantly associated with TNM stage, further supporting its potential role in PDAC progression. These data are consistent with previous reports linking *CLDN1* and *CLDN4* with epithelial disorganization and enhanced motility in aggressive cancers, including PDAC.^56,57^

The prognostic significance of Claudin expression was confirmed through Cox regression and Kaplan – Meier survival analyses, revealing that elevated *CLDN1* and *CLDN4* levels predicted poor overall survival, whereas high *CLDN5* and *CLDN15* expression correlated with a favorable prognosis.^58^ Importantly, multivariate analysis confirmed that Claudin expression retained independent prognostic value beyond traditional clinicopathological parameters. Such findings suggest that Claudins, particularly *CLDN1* and *CLDN4*, may serve as clinically relevant biomarkers for PDAC risk stratification and prognosis.

Our immune deconvolution analysis provided further insights into the potential immunomodulatory role of Claudins. In tumors with low *CLDN1* and *CLDN4* expression, there was a positive correlation with B cells, cancer-associated fibroblasts (CAFs), and cytotoxic CD8^+^ T cells, indicating a permissive microenvironment for immune infiltration. In contrast, high *CLDN1* and *CLDN4* expression was associated with decreased infiltration of most immune cell subsets, suggesting immune evasion or exclusion. Interestingly, *CLDN1* retained a weak positive correlation with CD8^+^ T cells even in high-expression tumors, suggesting context-dependent regulation of immune accessibility. This duality implies that Claudin expression not only influences epithelial integrity but may also dictate immune landscape remodeling within the PDAC microenvironment.

Crucially, our experimental data (Figure 7) provide functional validation of these transcriptomic observations. Using CRISPR/Cas9-mediated *CLDN1* knockout in Capan-1 cells, which normally express high endogenous *CLDN1*, we observed a significant reduction in proliferation, as measured by PrestoBlue assays. *CLDN1*-deficient cells also displayed markedly reduced migratory potential in wound-healing assays, confirming that *CLDN1* supports cell motility and metastatic behavior. Furthermore, Western blot analysis revealed that *CLDN1* knockout suppressed the EMT transcription factors Snail and Slug, while upregulating E-cadherin and downregulating vimentin. These molecular changes indicate a reversal of EMT and restoration of epithelial phenotype, consistent with the hypothesis that *CLDN1* functions as a pro-metastatic factor in PDAC. Together, these results confirm that the elevated *CLDN1* expression observed in patient datasets is not merely correlative but functionally contributes to PDAC aggressiveness.

Integrating these computational and experimental findings, it is evident that *CLDN1* plays a dual role in PDAC – structural and signaling. While normally maintaining epithelial cohesion, aberrant overexpression or mislocalization of *CLDN1* disrupts cell polarity, enhances cell plasticity, and activates EMT pathways. Through its interaction with EMT transcription factors and its influence on the immune milieu, *CLDN1* emerges as a nodal regulator linking epithelial barrier breakdown to immune escape and metastasis. This aligns with growing evidence that tight junction proteins act as dynamic participants in oncogenic signaling rather than passive structural elements.

Beyond its prognostic significance, targeting Claudin-1 therapeutically represents an important future direction for PDAC management. Given its cell-surface localization and frequent overexpression in tumors, *CLDN1* is an attractive candidate for monoclonal antibody – based therapies, including neutralizing antibodies or antibody – drug conjugates designed to selectively eliminate Claudin-1–high tumor cells. Small-molecule inhibitors that disrupt Claudin-1–mediated signaling interactions or interfere with tight junction-associated oncogenic pathways may also provide novel treatment strategies. Furthermore, considering our findings linking *CLDN1* expression with immune exclusion, therapeutic targeting of Claudin-1 could potentially enhance immune cell infiltration and improve responses to immune checkpoint inhibitors. Combination strategies integrating Claudin-1 targeting with chemotherapy or immunotherapy may therefore offer a more effective and personalized treatment approach for PDAC patients. Future preclinical and clinical studies are warranted to evaluate the feasibility and safety of these strategies.

In conclusion, this study provides a comprehensive overview of the Claudin family in PDAC by integrating bulk and single-cell transcriptomic data with experimental validation. The findings establish *CLDN1* and *CLDN4* as key oncogenic Claudins driving tumor progression and poor prognosis, while *CLDN5* and *CLDN15* may play protective roles. The newly generated in-vitro evidence demonstrates that *CLDN1* loss suppresses proliferation, migration, and EMT, providing a direct mechanistic link between Claudin expression and tumor behavior. Collectively, these results underscore Claudins as promising prognostic biomarkers and potential therapeutic targets in PDAC. Future work should explore Claudin-directed therapeutic strategies such as monoclonal antibodies or small molecules to disrupt tumor-promoting Claudin signaling and restore epithelial integrity, thereby improving therapeutic outcomes for PDAC patients.

Immune cell proportions were estimated using deconvolution-based algorithms. Patients were stratified into low- and high-expression groups using the top and bottom quartiles (Q1 vs. Q4) as cutoffs. Correlation values represent Pearson’s correlation coefficients, with the direction and magnitude indicating positive or negative associations between Claudin expression and immune cell infiltration.

## Supplementary Material

Supplemental Material
