# Supplementary material for: Integrated transcriptomic and functional characterization of Claudin-1 reveals its oncogenic and immunomodulatory roles in pancreatic ductal adenocarcinoma
Source: Tissue Barriers. 2026 Apr 14;14(3):2648150. doi: 10.1080/21688370.2026.2648150 (PMC13540116; doi:10.1080/21688370.2026.2648150)
Supplement: Supplementary file 1 [file KTIB_A_2648150_SM7035.docx]

**Table 1.** List of primary antibodies that have been used in the present study.

| S. No. | Name | Molecular weight (kDa) | Catalog No. | Manufacturer |
| --- | --- | --- | --- | --- |
| 1. | E-cadherin | 120 | 20874-1-AP | Proteintech® |
| 2. | Vimentin | 65 | #5741 | Cell signaling technologies |
| 3. | Claudin-1 | 21 | 13050-1-AP | Proteintech® |
| 4. | Snail (SNAI) | 29 | sc-271977 | Santa Cruz Biotechnology Inc. |
| 5. | Slug | 31 | sc-166476 | Santa Cruz Biotechnology Inc. |
